# Effect of Solvent on the Hydrodynamic Properties of Collagen

**DOI:** 10.3390/polym13213626

**Published:** 2021-10-21

**Authors:** Katarzyna Lewandowska, Marta Szulc, Alina Sionkowska

**Affiliations:** Department of Biomaterials and Cosmetics Chemistry, Faculty of Chemistry, Nicolaus Copernicus University in Toruń, Gagarin 7, 87-100 Toruń, Poland; reol@umk.pl (K.L.); marta.sz@doktorant.umk.pl (M.S.)

**Keywords:** collagen, collagen solutions, intrinsic viscosity, denaturation, UV irradiation

## Abstract

In this study, the effect of solvent on the hydrodynamic properties of collagen extracted from tail tendons of young rats was researched. Collagen was dissolved in various aqueous carboxylic acid solutions, including acetic acid (AA), acetic acid with the addition of sodium chloride (AA/NaCl), formic acid (FA), lactic acid (LA), citric acid (CA), and also citrate buffer at pH = 3.7 (CB). The properties of collagen solutions at a concentration of 0.45 mg/mL were characterized based on the viscometric method. The reduced viscosity, intrinsic viscosity, and Huggins coefficient of collagen solutions and effect of solvent, temperature, and UV irradiation on these properties were investigated. Collagen solutions in acetic acid, acetic acid/NaCl, and citrate buffer were irradiated with UV light up to 1 h, and the viscosity of collagen solutions was measured. It was found that the organic acids used as solvent affected viscosity behavior, denaturation temperature, and stability of collagen solutions. The lowest values of studied parameters were obtained for the collagen solutions in acetic acid with the addition of sodium chloride. Thus, the effect of various aqueous carboxylic acid solutions on collagen solutions properties and denaturation temperature can also be affected by the sodium chloride addition. The results of this research can be crucial for the preparation of collagen solutions for both cosmetic and biomedical applications.

## 1. Introduction

Collagen is a natural macromolecule isolated from natural sources such as mammal tendons and placenta; feet, skin, and sternal cartilage from domestic birds, for instance, chickens, turkeys, and ducks; bovine skin; and the tendons and bones of buffalos, lamb, rabbits, marine species, and others [1,2,3]. Due to its excellent biocompatibility, weak antigenicity, and controlled biodegradability, collagen is a primary biomaterial used in the food and cosmetic industries and the fields of tissue engineering and pharmacy. Some new materials based on collagen processing involve aqueous solutions. It is worth mentioning that the solubility of collagen in acidic pH depends on the age of the tissues, and it has a tendency to decrease with time due to a higher number of crosslinkers in older tissues. Collagen obtained from young tissue is more soluble than that extracted from maturated ones. It is not easy to compare solution behavior of various types of collagens. In the literature, one can find 29 types of collagens [1,4,5]. They differ in primary and secondary structures. Moreover, some of them are highly cross-linked and not soluble at all. Collagen is not a simple protein, and it is not possible to go to the general conclusion regarding solubility and the effect of solvent on the hydrodynamic properties of collagen of all types. Moreover, some collagens occur in tissues in a very small amount, so it is not easy to extract such collagens for laboratory investigation. The most widely used collagen in cosmetic and biomedical applications is collagen type I. This type of collagen is soluble in acidic pH, and its solubility depends on the age of the tissues from which collagen was extracted. Using collagen solution, one can obtain collagen films by solvent evaporation and 3D sponges by freeze-drying [4,5,6,7]. Collagen solutions are also used in aesthetic medicine as fillers. The subcutaneous injection of soluble collagen may improve the quality and density of the skin, repairing its dermatological defects [8]. Thus, the viscosity behavior of collagen solutions is essential for many applications. The viscosity behavior is also important in the characterization of collagen solution during the denaturation process. Non-denatured collagen gives a more viscous solution than a denatured one, so viscometric measurements are necessary when we carry out research using native collagen just to control its quality [9]. The viscometric properties of a collagen solution also depend on the acetic acid or other acids’ concentrations [10]. The interactions between collagen molecules and an acidic solvent are mainly by hydrogen bonds. The interactions by hydrogen bonds also occur between collagen molecules in concentrated collagen solutions. Collagen can be dissolved in several organic acids, but for cosmetic applications, mainly collagen solutions in lactic acid and citric acid are prepared. Lactic acid is also used in cosmetic formulas to gently exfoliate the skin, and it promotes moisturizing, evacuation of dead cells, and cell renewal. It is crucial to know the effect of various aqueous carboxylic acid solutions on collagen solutions properties, denaturation temperature of collagens, and the possibility to make emulsions containing collagens in temperatures higher than room temperature. Film-forming properties may also depend on the interactions between collagen molecules and molecules of the solvent. Preparation of 3D sponges by freeze-drying a collagen solution can also be influenced by the type of solvent and its interactions with collagens. Moreover, after freeze-drying a collagen solution, a small amount of solvent can be found in the sponge.

The purpose of this study was to investigate the effect of various solvents (AA, AA/NaCl, FA, LA, CA, and CB) on the hydrodynamic properties and denaturation temperature of collagen solutions. Viscometric studies have been used for many years for the characterization of natural and synthetic polymers in the dilute polymer solution [11,12,13,14,15]. This technique gives precious information regarding the viscosity, molecular weight, stability in different conditions, and others. We used a technique which is a simple and inexpensive method to characterize polymer molecules in the solution and to investigate the influence of various solvents on viscosity behaviors. The intrinsic viscosity [*η*] and Huggins coefficient *k_H_* are important information on the nature of the polymer in a solution, which characterizes the size and interaction of polymer chains. To the best of the author’s knowledge, the effect of various aqueous carboxylic acid solutions on collagen solutions by the viscometric method has not been investigated yet. We believe that the results can be useful in preparation of collagen solutions and gels. The poor water solubility of collagen seriously limits the application of collagen in fields such as injectable biomaterials and cosmetics.

## 2. Materials and Methods

### 2.1. Materials

Collagen was obtained in our laboratory from the tail tendons of young rats [16,17]. Briefly, in the first step, tail tendons were resected and washed in distilled water and dissolved in 0.1 mol/dm^3^ acetic acid for three days at 4 °C. After that, the prepared solution was then spun at 10,000 rpm for 10 min in a Sorvall centrifuge, and the soluble fractions were decanted. In the second step, the solution was frozen at −18 °C and lyophilized at −55 °C and 5 Pa for 48 h (ALPHA 1–2 LD plus, CHRIST, Osterode am Harz, Germany). All chemicals and reagents applied in this study were supplied by POCh (Avantor, Gliwice, Poland) and Chempur (Piekary Śląskie, Poland). These materials were of analytical grade and applied as received without further purification. IR spectroscopy was used to confirm that the lyophilizate contains collagen.

### 2.2. Solution Preparation

After lyophilization, the collagen solution was prepared by diluting lyophilizate in 0.1 mol/dm^3^ various aqueous solution including acetic acid (AA), formic acid (FA), lactic acid (LA), citric acid (CA), 0.1 mol/dm^3^ aqueous acetic acid/0.2 mol/dm^3^ aqueous NaCl (AA/NaCl), and also citrate buffer at pH = 3.7 (CB) at the 0.45 mg/mL concentration. The solution was shaken at regular intervals for 48 h and at 4 °C. After this time, the solution was clear. 

### 2.3. Viscometric Method

The viscometric measurements of dilute collagen solutions (*c* = 0.045 g/dL) in various solvents were carried out in a controlled thermostatic bath at 25 ± 0.1 °C using an Ubbelohde capillary viscometer with a viscometer constant of 31.34 s^2^. Distilled water was applied as the calibration liquid for an Ubbelohde viscometer in the temperature range between 22 °C and 32 °C. Before measurements, each collagen solution was filtered through G1 sintered glass filter. The stock collagen solution was prepared and diluted, producing the four lower concentrations made through the addition of an appropriate amount of solvent to the collagen solution. The flow time of solution was taken as the average of three readings with an accuracy of ± 0.01 s. The reduced viscosity of collagen solution was calculated by Equation (1):

(1) $$\frac{\eta_{sp}}{c}=\frac{t-t_{0}}{t_{0}c}$$

where $\frac{\eta_{sp}}{c}$ is the reduced viscosity (dL/g), *t* is the flow time of the collagen solution (s), *t*_0_ is the flow time of the solvent (s), and *c* is the concentration of collagen solution (g/dL). The intrinsic viscosity was determined from the plot of reduced viscosity vs. collagen concentration using the classical Huggins equation [18,19,20], shown in Equation (2):

(2) $$\frac{\eta_{sp}}{c}=\left[\eta \right]+k_{H}{\left[\eta \right]}^{2}c$$

where [*η*] is the intrinsic viscosity (dL/g) and *k_H_* is the Huggins coefficient (dimensionless). The plot of reduced viscosity vs. concentration provided a straight line, where interception and slope are, respectively, equal to [*η*] and *k_H_*[*η*]^2^. Kinetic energy correction was taken into account for the evaluation of the intrinsic viscosity.

The viscosity average molecular weight of collagen (${\bar{M}}_{v}$) was calculated by its intrinsic viscosity in citrate buffer at pH = 3.7 using the Mark–Houwink–Sakurada equation [19,20], as follows:

(3) $$\left[\eta \right]=K{\bar{M}}_{v}^{a}$$

where *K* and *a* are empirical viscometric constants which depend on the kind of polymer and solvent, and also on temperature. Collagen in citrate buffer at pH = 3.7 had the constants of K = 1.23 × 10^−9^ dL/g and a = 1.80 at 25 °C [21]. 

### 2.4. Determination of Denaturation Temperature by Viscometric Method

For this measurement, the solutions of 0.5 mg/mL collagen were prepared in 0.1 mol/dm^3^ acetic acid (AA), 0.1 mol/dm^3^ aqueous acetic acid/0.2 mol/dm^3^ aqueous NaCl (AA/NaCl) and citrate buffer at pH = 3.7 (CB). The thermal denaturation plots were obtained from 25 °C to 45 °C, and temperature was raised stepwise and maintained for 20 min. All the experiments were carried out till three subsequent readings in the series reached a constant value. Plots of reduced viscosity as a function of temperature were made for the collagen solutions. The thermal denaturation temperature was expressed as a midpoint temperature between the extrapolated line for stock collagen solution and that for fully denatured collagen solution on the reduced viscosity vs. temperature plot [15]. 

### 2.5. UV Irradiation of Dilute Collagen Solutions

The collagen solution with a concentration of 0.5 mg/mL in 0.1 mol/dm^3^ acetic acid (AA) and citrate buffer at pH = 3.7 (CB) were irradiated using a UV lamp, ULTRAVIOL NBV 15, which emitted mainly UVC with 254 wavelength for different time intervals (0–60 min). The intensity of UV light was 21.5 W/m^2^. Collagen solutions were irradiated 5 cm from the UV lamp. After that, the viscosity behavior was measured using the same Ubbelohde capillary viscometer at 25 ± 0.1 °C. Before measurements, the collagen solution was filtered through G1 sintered glass filter. The flow time of solution was taken as the average of three readings with an accuracy of ±0.01 s. Plots of reduced viscosity as a function of time of UV irradiation were made for the collagen solutions.

## 3. Results and Discussion

### 3.1. Viscometric Studies

**Figure 1:**
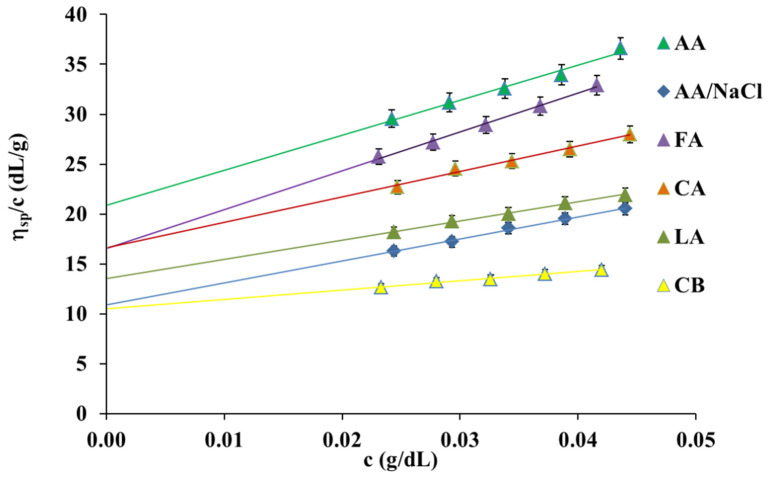
Reduced viscosity (*η_sp_*/*c*) values of collagen solution in different solvents at 25 °C.

The influence of solvents on the reduced and intrinsic viscosity of the collagen solutions at 25 °C was measured. Figure 1 presents the plots of the reduced viscosity vs. the concentration for the collagen solutions in six aqueous solvent solutions, including acetic acid (AA), acetic acid with the addition of sodium chloride (AA/NaCl), formic acid (FA), lactic acid (LA), citric acid (CA), and also citrate buffer at pH = 3.7 (CB). It can be seen that all plots showed a linear relationship. The intercept of these plots corresponds to the intrinsic viscosity. [*η*] values, and comparisons between the different solvents used in collagen solution, are listed in Table 1.

As shown in Figure 1 and Table 1, the collagen solution in AA had the highest reduced and intrinsic viscosity while the collagen solution in AA/NaCl and CB had the lowest. This behavior was due to the addition of salt to the aqueous carboxylic acid solution (AA and CA). The addition of salt to the collagen solutions causes the increase in the ionic strength in the solution and screens the electrostatic charges. As a consequence, a decrease in the reduced and intrinsic viscosity in AA/NaCl and CB solutions in comparison with that of the other solvents is observed (Figure 1 and Table 1). It is well known that the intrinsic viscosity can be applied as a measure of the solvent power [20,22]. The larger value of intrinsic viscosity indicates more powerful interactions between the polymer molecules and the solvent and a better solvent for the polymer. Thus, AA is an excellent solvent for collagen, and formic acid is better than lactic acid and also citrate buffer. In citrate buffer and AA/NaCl, the intrinsic viscosity decreased significantly compared to other solvents. The Huggins coefficient was also determined from Equation (2) and is also listed in Table 1.

The parameter *k_H_* describes interactions between various polymeric molecules present in the solutions. The Huggins coefficient decreases with the solvent power and for polymers in thermodynamically good solvents usually falls in the range 0.2–0.4. Huggins parameter is not constant for chosen polymer–solvent systems, but depends on temperature and molecular weight [18,20,23]. Thus, the *k_H_* value should be lower in good solvents and at higher molecular weights. There are, however, polymer–solvent systems where Huggins coefficient is significantly higher than 0.5 [23]. This is attributed to the association of polymer chains and the formation of molecular aggregates. According to the value of Huggins coefficient, AA and CB are good solvents for collagen. Citrate buffer at pH = 3.7 is used for the determination of the viscosity average molecular weight of collagen in the literature [21] and in this study. The highest value of *k_H_* we found for the collagen solution in AA/NaCl. It is due to the association of collagen chains after the addition of NaCl to the AA solution.

For the collagen solution in CB, the intrinsic viscosity was found to be 10.52 ± 0.19 dL/g. Hence, the viscosity average molecular weight for collagen used in this study was calculated, and its value was 329 kDa. This result is consistent with other reports [24,25], which indicate that a standard collagen molecule is typically 300 kDa where each of the alpha stands is approximately 100 kDa.

### 3.2. Determination of Denaturation Temperature by Viscometric Method

**Figure 2:**
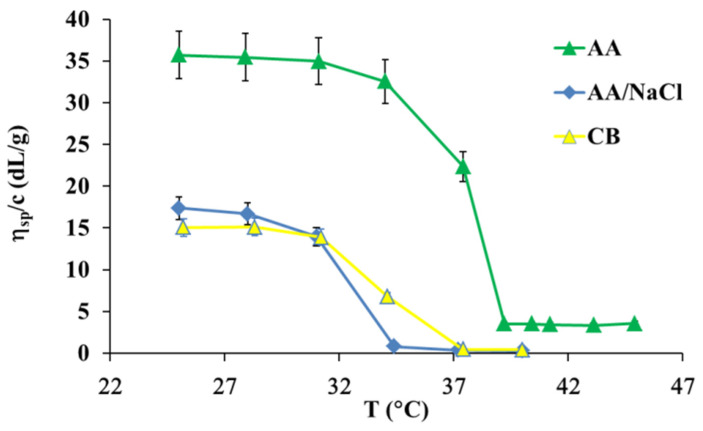
Plots of reduced viscosity vs. temperature for 0.5 mg/mL collagen solutions in three solvents.

Figure 2 shows the thermal denaturation plot of collagen solutions.

The denaturation temperature of collagen dissolved in three aqueous solvent solutions such as AA, AA/NaCl, and CB was calculated using temperature induced change in the reduced viscosity plot. As shown in Figure 2, the reduced viscosity of the collagen solution in AA decreased as temperature increased from approximately 35 °C to 40 °C, while the reduced viscosity decreased from approximately 31 °C to 37 °C for the collagen solution in CB and from approximately 31 °C to 34 °C the collagen solution in AA/NaCl. Thus, the denaturation temperature is the biggest for collagen in acetic acid solution and it changes as T_d_ AA (38 °C) > T_d_ CB (35 °C) > T_d_ (33 °C) AA/NaCl. The addition of salt to the aqueous acidic solution of collagen decreases the thermal stability of the collagen triple helix in the solution. After the addition of salt, the collagen molecules conformation reduces to the statistical coil conformation. The helix to coil transition of salt treated collagen depends on the degree of hydration and the additive concentration. According to the literature data and results of this study, the viscosity of collagen solution decreases with the addition of the denaturing agents, and it depends on the additive concentration [26].

### 3.3. Influence of UV Irradiation on Dilute Collagen Solutions

**Figure 3:**
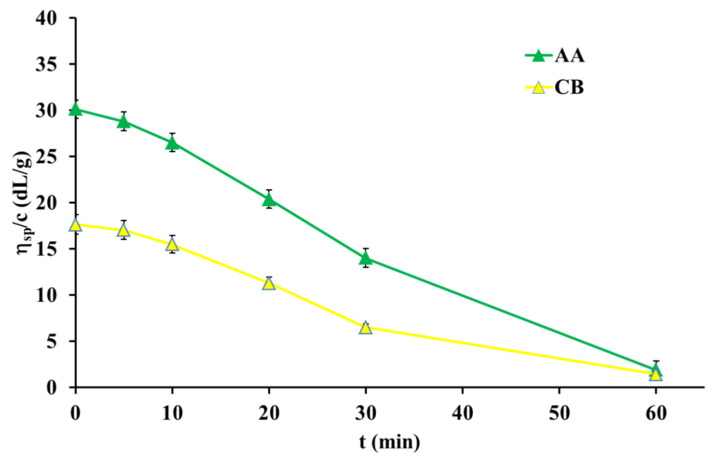
Plots of reduced viscosity vs. times of UV irradiation for 0.5 mg/mL collagen solutions.

Figure 3 shows the reduced viscosity as a function of time of UV irradiation for the collagen solution in AA and CB. The addition of NaCl to the aqueous acidic solution of collagen leads to the decrease in the thermal stability of collagen triple helix in the solution (as shown in Figure 2), and for this reason the irradiation of collagens in already partially denatured collagen solution in acetic acid, with the addition of sodium chloride (AA/NaCl), was not conducted.

As shown in Figure 3, the plot shapes are similar, and the curves are only shifted relatively to each other. It is clear that the reduced viscosity of collagen solution decreased by approximately 10% when the time of irradiation increased from 0 to 10 min. A time increase of 10 min leads to the approximately 30% viscosity drop. After one hour of irradiation, the viscosity of the collagen solution decreased by 93%. This suggests that after one hour of UV irradiation, collagen molecules are fully denatured. Thus, both collagen solutions showed a similar sensitivity to UV irradiation. It is well known that UV irradiation leads to partial cleavage of the hydrogen bonds responsible for the ternary structure of collagen [27].

## 4. Conclusions

In this study, the effect of various solvents (AA, AA/NaCl, FA, LA, CA, and CB) on the hydrodynamic properties and denaturation temperature of collagen solutions were examined. It was found that the kinds of solvents show a great influence on viscosity behavior and denaturation temperature. According to the intrinsic viscosity and Huggins coefficient values, AA is a very good solvent for the collagen molecules. For the collagen solution in CB, the intrinsic viscosity was found to be 10.52 ± 0.19 dL/g. The denaturation temperature is maximum for collagen solution in acetic acid, and it changes as T_d_ AA (38 °C) > T_d_ CB (35 °C) > T_d_ (33 °C) AA/NaCl. Moreover, both collagen solutions in AA and CB showed similar sensitivity to UV irradiation. The results of this research can be useful for preparation of collagen solution for several applications, as the choice of the solvent and pH of solution may influence the collagen stability and performance.

## Figures and Tables

**Table 1:** Values of intrinsic viscosity [*η*] and Huggins coefficient *k_H_* for collagen solutions in different solvents at 25 °C.

Solvent	[*η*] dL/g	*k_H_*	R^2^
AA	20.90 ± 0.90	0.80	0.992
AA/NaCl	10.88 ± 0.34	1.87	0.997
FA	16.55 ± 0.45	1.42	0.998
CA	16.58 ± 0.64	0.93	0.993
LA	13.83 ± 0.16	0.96	0.999
CB	10.52 ± 0.19	0.84	0.995

## Data Availability

Data is contained within the article.
